# Variation in Apical Hook Length Reflects the Intensity of Sperm Competition in Murine Rodents

**DOI:** 10.1371/journal.pone.0068427

**Published:** 2013-07-03

**Authors:** Martin Šandera, Tomáš Albrecht, Pavel Stopka

**Affiliations:** 1 Biocev group, Department of Zoology, Faculty of Science, Charles University in Prague, Prague, Czech Republic; 2 Institute of Vertebrate Biology, Academy of Sciences of the Czech Republic, Brno, Czech Republic; Uppsala University, Sweden

## Abstract

**Background:**

Post-copulatory sexual selection has been shown to shape morphology of male gametes. Both directional and stabilizing selection on sperm phenotype have been documented in vertebrates in response to sexual promiscuity.

**Methodology:**

Here we investigated the degree of variance in apical hook length and tail length in six taxa of murine rodents.

**Conclusions:**

Tail sperm length and apical hook length were positively associated with relative testis mass, our proxy for levels of sperm competition, thus indicating directional post-copulatory selection on sperm phenotypes. Moreover, our study shows that increased levels of sperm competition lead to the reduction of variance in the hook length, indicating stabilizing selection. Hence, the higher risk of sperm competition affects increasing hook length together with decreasing variance in the hook length. Species-specific post-copulatory sexual selection likely optimizes sperm morphology.

## Introduction

Sperm competition and other postcopulatory processes have been considered as important evolutionary selective forces [1], [2], [3]. Sperm competition is reflected by epigamic behaviour and morphology of animals [4], [5], [6], [7] and also by behaviour and morphology of sperm cells [8], [9], [10], [11], [12], [13], [14]. It has been shown that some sperm traits indicate the risk of sperm competition [9] and could be influenced by directional [11], [15] and/or stabilizing selection [11], [16].

Species with higher risk of sperm competition tend to have longer and faster sperm - e.g. [4], [17], [14]. However, phylogenetically controlled analysis of sperm variables across mammals and birds did not confirm a clear association between sperm competition and sperm length [18], [15]. On the other hand, sperm competition acts to reduce between-male and within-male variation in sperm length in various animal taxa, including birds [19], [12], [16] and hymenopterans [13], possibly through an increase in strength of post-copulatory selection on sperm traits [20], [11]. Sperm length variation has been found as a good indicator of extrapair paternity (i.e. the risk of sperm competition) in passerine birds [16] and is recently used as a proxy measure for sperm competition in comparative studies [21].

Sperm heads in many rodent species possess apical hooks [22], [23], [24], [25]. The apical hook is an important structure for linking sperm into aggregations (‘trains’), each consisting of hundreds of cells [10]. Sperm cooperation by forming trains has been presented as an advantageous strategy in sperm competition and is considered as a main adaptive mechanism in sperm competition in rodents [8], [9], [10]. It has been shown that species with higher risk of sperm competition have sperm with longer apical hooks [9] and the hooks are more flexible [26]. However, whether postcopulatory sexual selection decreases the variation in hook size and sperm cell size in rodents remains unclear.

**Figure 1:**
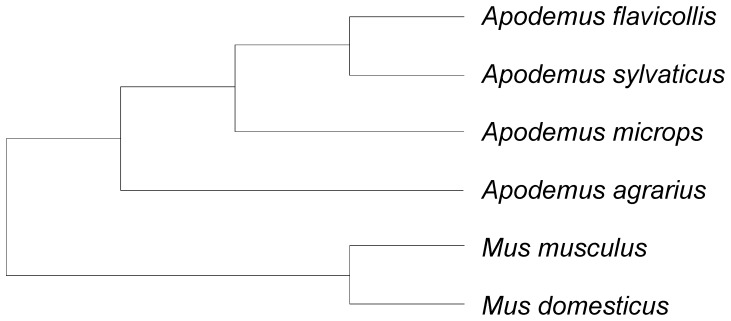
Phylogeny of studied species. The figure illustrates the phylogenetic relationships among the studied species [40], [41], [42].

This study aims at elucidating the influence of the risk of sperm competition as an evolutionary force of stabilizing selection on sperm morphology. In six rodent species (see Figure 1) we investigated whether the species with higher risk of sperm competition possess lower variance in sperm hook length and tail length in a phylogenetically controlled analysis. Since relative testis weight is a good indicator of promiscuity [9], [27], this measure also reflects the risk of sperm competition. We hypothesized that in more promiscuous species, due to a higher risk of sperm competition, the mean values of sperm traits (i.e. hook length, sperm tail length) are higher but the between male and within male variances of these characters are lower.

## Results

Both measured sperm traits (i.e. hook length and tail length) differed significantly between the species (Anova, p<0.001; hook length F(5,46) = 56.01; tail length F(5,46) = 27.19). Coefficients of between and within-male variation (hook length: CV_bm hook_ and CV_wm hook_, tail length: CV_bm tail_ and CV_wm tail_) in sperm traits (Table S1) revealed low overall variance in the tail length. The most variable hook length was found in *M. domesticus*, the least variable in *A. agrarius* (in case of CV_bm_) and in *A. sylvaticus* (in case of CV_wm_). Sperm of *M. musculus* showed the largest variation in the tail length and the relative testis weight (i.e. our proxy measure for sperm competition) differed significantly between the species studied (Anova, F(5,46) = 88.684, p<0.001). The highest relative testis weights were observed in *A. sylvaticus* and followed by *A. agrarius*. The lowest testis weights were detected in *M. musculus* and by *M. domesticus* (Table S1).

**Figure 2:**
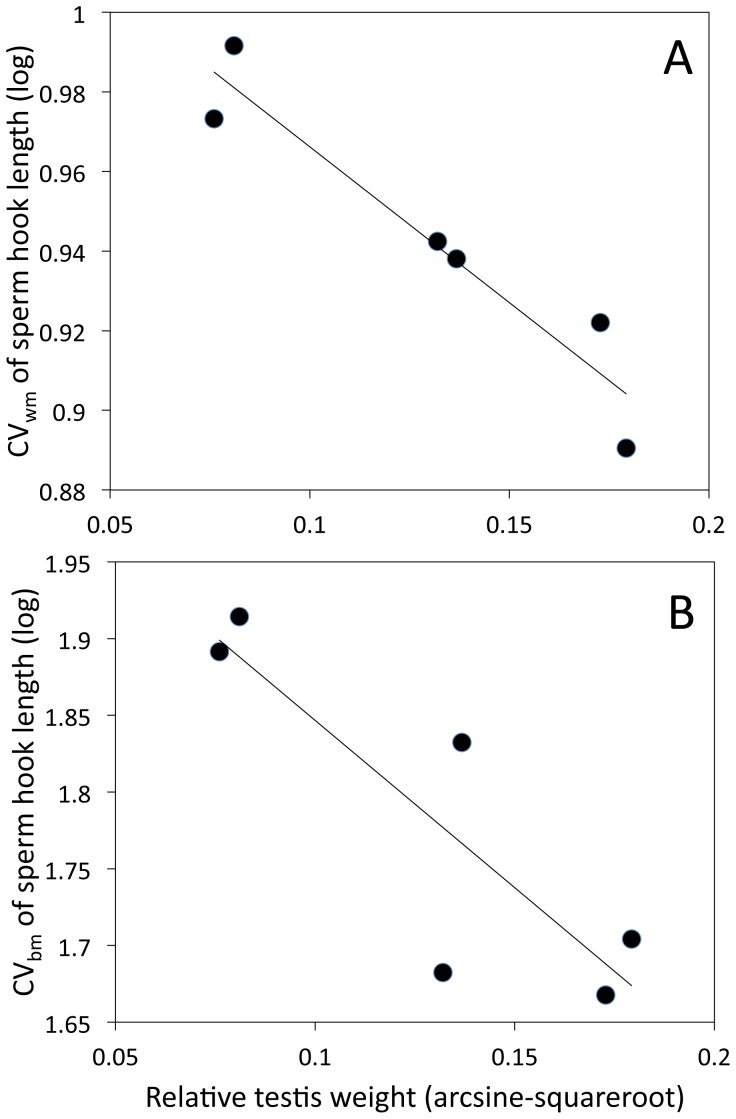
Relationships between relative testis weight and variation in sperm hook length in murine rodents. A) Relationship between relative testis weight and within-male variation in sperm hook length. The figure illustrates the linear regression of the coefficient of within-male variation in apical hook length on the proportion of relative testis weight for six studied rodent species. B) Relationship between relative testis weight and between-male variation in sperm hook length. The figure illustrates the linear regression of the coefficient of within-male variation on the proportion of relative testis weight in apical hook length for six studied rodent species.

The mean hook length, and both CV_wm hook_ and CV_bm hook_, were strongly associated with sperm competition (Table 1A). Hook length increased and variation in this trait decreased with increasing sperm competition (see slopes and associated SE in Table 1A). Relative testis mass explained 88% variation in case of CV_wm hook_, 82% in case of CV_bm hook_ and 94% variation in case of hook length (Table 1A, Figure 2).

**Table 1:** Generalised linear least square tests of: a) the effect of the relative testis weight (proxy measure for post-copulatory sexual selection) on sperm length traits, b) the effect of hook length on its variation c) the effect of sperm tail length on its variation.

Sperm trait	Slope ± SE	t-value	p	λ	R^2^
a) relationships between relative testis weight and sperm traits					
hook length	25.6±2.96	8.63	<0.001	<0.001^1.0, 0.14^	0.94
CV_wmhook_	−1.70±0.28	−6.07	0.004	<0.001^1.0, 0.13^	0.88
CV_bmhook_	−5.31±1.25	−4.26	0.013	<0.001^1.0, 0.11^	0.82
tail length	95.5±36.9	2.59	0.061	0.999 ^0.17, 1.0^	0.53
CV_wmtail_	−0.21±0.78	−0.27	0.804	<0.001^1.0,0.13^	0.017
CV_bmtail_	0.03±3.90	0.008	0.994	<0.001^1.0, 0.09^	<0.001
b) relationships between hook length and hook variation					
CV_wmhook_	−0.062±0.014	−4.27	0.013	<0.001^1.0, 0.06^	0.82
CV_bmhook_	−0.212±0.035	−6.05	0.004	<0.001^1.0, 0.07^	0.90
c) relationships between tail length and tail variation					
CV_wmtail_	0.001±0.009	0.036	0.97	<0.001^1.0, 0.11^	<0.001
CV_bmtail_	0.002±0.043	0.054	0.96	<0.001^1.0, 0.09^	<0.001

CV_wm_ is the average coefficient of within-male variation in hook length and tail length. CV_bm_ is the coefficient of between-male variation in sperm traits, adjusted for sample size (see Methods). The statistical analyses were performed in R and based on transformed variables to approach normality (arcsine square-root for the proportion of relative testis weight, log for CV_wm_ and CV_bm_). Slopes were tested against the prediction of 0 using the *t*-test. Lambda (λ) indicates the level of phylogenetic dependence in the data, with superscripts giving p-values for test of λ = 0 and λ = 1 respectively. R^2^ values indicate the proportion of total variance explained.

Compared to sperm hook, the associations between sperm competition and sperm tail length were much weaker. Although testes mass explained as much as 53% variation in the tail length, this relationship was not significant (p = 0.061, Table 1b) and there was no association between sperm competition and CV_bm tail_ and CV_wm tail_ (Table 1a).

The hook length was negatively correlated with its variation (both CV_wm hook_ and CV_bm hook_; Table 1b). This relationship was significant (CV_wm hook_ p = 0.013 and CV_bm hook_ p = 0.004; 82% and 90% variation explained, Table 1b). There was no significant relationship between the tail length and its variation (Table 1c).

## Discussion

Our data confirmed previous findings that have demonstrated important role of post-copulatory sexual selection in evolution of apical hook length in murine rodents. Across murine rodents, species with higher risk of sperm competition possess longer apical hooks [9], [26], and this is well supported also in the 6 species selected in our study. In addition, we demonstrate that sperm competition also affects the between and within male variation in the hook length. In agreement with the idea of stabilizing post-copulatory sexual selection on sperm traits [20] we have documented that higher risk of sperm competition, as expressed by relative testis weight, leads to reduced variance in the hook length. The analysis has also shown that the variance in hook length is decreasing with the increasing hook length.

The idea that higher risk of sperm competition is associated with reduced variation in sperm length, which has been documented in passerine birds [19], [12], [16], [28] and insects [13], seems unsupported in rodents. In murine rodents it is probably the hook morphology that determines fertilization success of sperm cell (i.e. affecting ability of sperm cells to cooperate), while stabilizing selection for optimal sperm length might be less important than in organisms with specialized female sperm storing organs like birds and hymenopterans. However, it should be noted that although our analysis did not confirm the association between variance in the tail length and sperm competition, the variance in the tail length was low in the studied species generally. This may indicate that even in house mice sperm competition may to some extent play a role and has reduced the variance in the tail length. The house mice compared with the field mice possess smaller testes and about two to three times lower rate of multiple paternity [27], but the multiple paternity rates in *M. domesticus*, 23% [29] and 26% [30] suggest a certain degree of promiscuity in house mice. Although *M. domesticus* in our samples had slightly higher relative testes mass than *M. musculus*, the hook length variation indicates higher levels of sperm competition in *M. musculus*. Detailed information about multiple paternity levels is needed for both house mouse subspecies to confirm this finding.

The apical hook morphology should point to different sperm strategies in sperm cooperation [9]. Our results are consistent with the described sperm behaviour. Sperm in the wood mouse (*Apodemus sylvaticus*) form aggregations (trains) that move faster than separate sperm cells [9], [10], while in the house mouse the trains are slower than the particular spermatozoa [9]. The post-copulatory sexual selection seems to be stronger in field mice than in house mice [27], [29] and may penalize sperm cells bearing suboptimal hooks. Directional selection may thus act so that sperm have longer apical hooks while stabilizing selection reduces variation in the hook length. Field mice of the genus *Apodemus* produce sperm with longer and uniform hooks, so they form more effective trains. Contrary to that, sperm in house mice have shorter and variable hooks, so they form less effective trains.

Within field mice, *A. agrarius* and *A. sylvaticus* may be considered as species with high level of the risk of sperm competition and with high level of promiscuity. This fact was indicated by sperm with long apical hooks and the least variance in the hook length (Table S1). It is consistent with the data on relative testis size and multiple paternity rates [27]. Compared with previous two species, *A. flavicollis* and *A. microps* showed shorter hooks and higher variances (CV_wm hook_ and CV_bm hook_) in the hook length. Surprisingly, the tails in *A. flavicollis* and *A. microps* were shorter than in house mice, on average, but the relative testis weight (Table S1) and the multiple paternity rates [31], [27], [29] indicated stronger effect of sperm competition in these field mice.

Different variations in hook length between field mice and house mice interestingly match with different rates of acrosomal reaction [32] and the related finding that the sperm in field mice do not express membrane cofactor protein CD46 [33], [34], [32]. Accelerated acrosome reaction of sperm in field mice allowing rapid fertilization proved to be advantageous in promiscuous species [32]. Sperm in field mice may then be protected against complement-mediated injury in female genital tract by other complement regulatory proteins, CD55 and CD59 [34].

The association between the relative testis weight and the tail length was quite strong, even though not significant (p = 0.06). This finding may partly support the hypothesis that species with higher risk of sperm competition have longer sperm [4], [17]. Although this hypothesis has been confirmed in some taxa (e.g., [14]), it was not confirmed by results of a comprehensive analysis across mammals [18]. The influence of sperm competition on tail length might be different in different groups of mammals. Data from passerine birds also suggest that the relationship between sperm competition and sperm length is not straightforward, with different directions of the relationship found in different passerine families [15].

We conclude that sperm competition in murine rodents affects mainly apical hooks and stabilizing selection causes reduced variance in the hook length in species with higher risk of sperm competition. Both coefficients of variation (CV_wm_ and CV_bm_) hold a great potential for use in further studies on sperm competition in different animal groups, but traits important in post-copulatory sexual selection for particular taxonomical groups should be identified. Unlike in passerines and some insects [19], [12], [13], the variation in sperm tail length was not associated with sperm competition in murine rodents.

## Methods

### Ethical Standards

All animal procedures were carried out in strict accordance with the law of the Czech Republic paragraph 17 no. 246/1992, and Animal Scientific Procedures paragraph 11, no. 207/2004, and the local ethics committee of the Faculty of Science of Charles University in Prague specifically approved this study in accordance with accreditation no. 24773/2008-10001. Animals were sacrificed by cervical dislocation.

### Mice

Fifty-two males of six species were used, four field mice species: herb field mouse *Apodemus microps* Kratochvíl et Rosický, 1952 ( = *A. uralensis* (Pallas, 1811), locality: Drnholec, Czech Republic), striped field mouse *Apodemus agrarius* (Pallas, 1771) (locality: Šebastovce, Slovakia), field mouse *Apodemus sylvaticus* (Linnaeus, 1758) (locality: Prague, Czech Republic), yellow-necked field mouse *Apodemus flavicollis* (Melchior, 1834) (locality: Drnholec, Czech Republic), two house mice species: eastern house mouse *Mus musculus* Linnaeus, 1758 (locality: Sedlečko, Czech Republic), western house mouse *Mus domesticus* Schwarz et Schwarz, 1943 (locality: Straas, Germany).

Age of individuals used in experiments was unknown because it is difficult to infer the age of rodents captured in the field. Although, sperm traits may potentially be affected by age in rodents [35] we only used fully-grown males in reproductive stage in this study with no obvious signs of senescence or immaturity (e.g. weight of individuals was close to the population mean). Mice were maintained in a pathogen-free facility for wild mice at the Department of Zoology, Faculty of Science, Charles University in Prague, Czech Republic.

### Data Collection and Preparation

Sperm from *cauda epididymis* were studied. Sperm samples were obtained by placing the incised *cauda epididymis* into microtubes with PBS (pH 7.34) for 5 minutes at 37°C under 5% CO_2_. The sperm suspension was then placed onto clean microscopic slides and smeared. Smears were air-dried and then fixed in methanol (8 min at −18°C) and in acetone (6 min at −18°C). Sperm were evaluated under a light microscope (Olympus BX 51, 600×magnification).

Only morphologically normal sperm were measured as reported [36] – we carefully inspected sperm smears and avoided just few broken sperm cells and cells with missing hook from analyses. The sperm apical hook length and tail (flagellum) length were measured using analySIS (Soft Imaging System) software. The sperm dimensions were assessed by the measurement method described by [9]. Thirty spermatozoa were analysed from each male. In total, we analysed 1560 spermatozoa in 52 males. Males and testes were weighed and the relative testis weight calculated. All measurements were conducted by only one person (M.S.).

As a standardized measure of variation, we used the coefficient of variation (CV = SD/mean×100), denoted as CV_bm_ for the between-male CV in mean length of sperm traits and CV_wm_ for the mean within-male CV in length of sperm traits. As CV will be underestimated for small sample sizes, we corrected CV_bm_ according to the formula: Adjusted CV_bm_ = (1+1/4*n*)×CV_bm_ [37]. Descriptive statistics of measured traits and their variation are given for each species in Table S1. We calculated and throughout the paper refer to coefficients of between and within-male variation in apical hook length (CV_bm hook_ and CV_wm hook_) and sperm tail (CV_bm tail_ and CV_wm tail_).

Statistical analyses: We applied a generalized least squares regression method in a phylogenetic framework [38], [39] with the phylogeny of species shown in Figure 1 [40], [41], [42] to evaluate the prediction that sperm traits reflect the strength of post-copulatory sexual selection in murine rodents. We used relative testis mass as our proxy for the strength of post-copulatory sexual selection in murine rodents. We have previously shown that the relative testis size is strongly associated with levels of multiple male mating in field mice (R^2^ = 0.836, slope = 20.05±4.96[SE] [27]. Constant branch lengths were assumed. We made univariate regressions for the three variables (total length, CV_wm_ and CV_bm_) for both sperm traits (sperm apical hook and tail) and the proportion of relative testis weight. To improve normality, all CVs were log transformed and the proportions of relative testis weight arcsine-squareroot transformed. The slopes were tested against the prediction of 0 using the t-test. For each test, an index of phylogenetic dependence, λ, was estimated, with values ranging between 0 (phylogenetic independence) and 1 (complete phylogenetic dependence), and tested with a likelihood ratio test against models with λ values set at 0 and 1. The analyses were performed in R [43] using the package APE [44] and a script provided by R. P. Freckleton, Department of Animal and Plant Sciences, The University of Sheffield.

## Supporting Information

Table S1 **Sperm hook length and tail length characteristics and relative testis weight in studied rodents.**
(DOCX)
