# Correction: Variation in Apical Hook Length Reflects the Intensity of Sperm Competition in Murine Rodents

**DOI:** 10.1371/annotation/e9485d81-a44b-456d-abee-fb331c6ad5fb

**Published:** 2013-10-23

**Authors:** Martin Šandera, Tomáš Albrecht, Pavel Stopka

A grant number and the initial provider of the funding for the grant from BIOCEV were incorrectly omitted from the Funding Statement. The Funding Statement should read: "This work was supported by the Academy of Sciences of the Czech Republic grant no. IAA601110908 (http://www.isvav.cz/projectDetail.do?row ​Id=IAA601110908). This publication is supported by the project 'BIOCEV - Biotechnology and Biomedicine Centre of the Academy of Sciences and Charles University - http://www.biocev.eu/en/' (CZ.1.05/1.1.00/02.0109), from the European Regional Development Fund. The funders had no role in study design, data collection and analysis, decision to publish, or preparation of the manuscript." 

