# Supplementary material for: Variation in Apical Hook Length Reflects the Intensity of Sperm Competition in Murine Rodents
Source: PLoS One. 2013 Jul 3;8(7):e68427. doi: 10.1371/journal.pone.0068427 (PMC3700964; doi:10.1371/journal.pone.0068427)
Supplement: Table S1 — Sperm hook length and tail length characteristics and relative testis weight in studied rodents. (DOCX) [file pone.0068427.s001.docx]

| species | n males  (n sperm) | hook length [µm]  mean ± SE | CV_wm_ | CV_bm_ | tail length  [µm]  mean ± SE | CV_wm_ | CV_bm_ | testes %  mean ± SE |
| --- | --- | --- | --- | --- | --- | --- | --- | --- |
| *A. agrarius* | 8 (240) | 7.5 ± 0.05 | 8.36 | 5.57 | 128.3 ± 0.16 | 1.44 | 1.42 | 2.96 ± 0.11 |
| *A. sylvaticus* | 6 (180) | 7.3 ± 0.05 | 7.77 | 6.05 | 122.3 ± 0.22 | 1.32 | 2.25 | 3.18 ± 0.19 |
| *A. flavicollis* | 9 (270) | 6.7 ± 0.05 | 8.67 | 8.14 | 117.7 ± 0.14 | 1.41 | 1.33 | 1.86 ± 0.43 |
| *A. microps* | 9 (270) | 7.0 ± 0.04 | 8.76 | 5.76 | 118.0 ± 0.14 | 1.49 | 1.43 | 1.73 ± 0.10 |
| *M. musculus* | 14 (420) | 5.0 ± 0.03 | 9.40 | 9.32 | 120.0 ± 0.17 | 1.55 | 2.48 | 0.58 ± 0.04 |
| *M. domesticus* | 6 (180) | 5.1 ± 0.05 | 9.81 | 9.82 | 119.6 ± 0.14 | 1.30 | 1.01 | 0.66 ± 0.01 |
